# Nanobody Therapeutics in Alzheimer’s Disease: From Molecular Mechanisms to Translational Approaches

**DOI:** 10.3390/antib15010001

**Published:** 2025-12-19

**Authors:** Deepika Godugu, Kranthi Gattu, Parul Suri, Abel B. Daartey, Krishna Jadhav, Satish Rojekar

**Affiliations:** 1 Department of Pharmacy, University College of Technology Sciences, Osmania University, Hyderabad 500007, Telangana, India; drdeepikagodugu@gmail.com; 2 Department of Pharmaceutical Sciences, College of Pharmacy and Health Sciences, St. John’s University, Queens, NY 11439, USA; kranthigattu.d@gmail.com; 3 Department of Biotechnology, Jaypee Institute of Information Technology, Noida 201309, Uttar Pradesh, India; parulsuri281291@gmail.com; 4 Department of Pharmacology, Faculty of Pharmacy and Pharmaceutical Sciences, Kwame Nkrumah University of Science and Technology, Kumasi AK-385-1973, Ghana; daarteyabel@gmail.com; 5 Institute for Bioengineering of Catalonia (IBEC), Baldiri Reixac 10-12, 08028 Barcelona, Spain; krishnajadhavphd@gmail.com; 6 Department of Pharmacological Sciences, Icahn School of Medicine, Mount Sinai, New York, NY 10029, USA

**Keywords:** amyloid-β and tau targeting, nanobodies, Alzheimer’s disease, brain delivery, therapeutics

## Abstract

Nanobodies (single-domain antibodies, VHHs) have emerged as versatile tools for evaluating and treating Alzheimer’s disease (AD). They offer distinct engineering benefits compared with traditional antibodies and small molecules, including small size, stability, and specificity. In AD, nanobodies have been shown in preclinical models to neutralize toxic amyloid-β oligomers, inhibit tau generation and aggregation, and modulate neuroinflammation, thereby demonstrating significant therapeutic potential. However, all nanobody applications in AD are discussed strictly as preclinical therapeutic potential rather than established clinical therapies, and direct clinical evidence in patients with AD is still lacking. Advanced engineering strategies, including intranasal and intrathecal routes, receptor-mediated transport, plasma protein binding with albumin, and focused ultrasound to facilitate brain penetration. Additionally, to improve nanobody delivery precision, half-life, and efficacy, strategies such as integrating nanobodies with nanoparticles, dendrimers, liposomes, and viral vectors are being employed. In fact, nanobodies are applied beyond monotherapy across multiple technological platforms to optimize brain delivery and target multiple targets. Nanobodies have been used on bispecific and trispecific antibody platforms, as well as in CRISPR/Cas9 editing and AI-driven technologies, to expand their applications. Recently, preclinical evidence has been mounting on the efficacy of nanobodies in clearing Aβ and tau, preserving synapses, and normalizing biomarkers. Comparison with FDA-approved anti-Aβ monoclonal antibodies (aducanumab, lecanemab, and donanemab) highlights opportunities and current translational gaps, including safety testing, half-life extension, and delivery optimization. This review critically delineates the current molecular mechanisms, emerging strategies, and delivery platforms, and emphasizes the potential of nanobodies as promising therapeutic and diagnostic molecules in AD therapeutics.

## 1. Introduction

**Figure 1:**
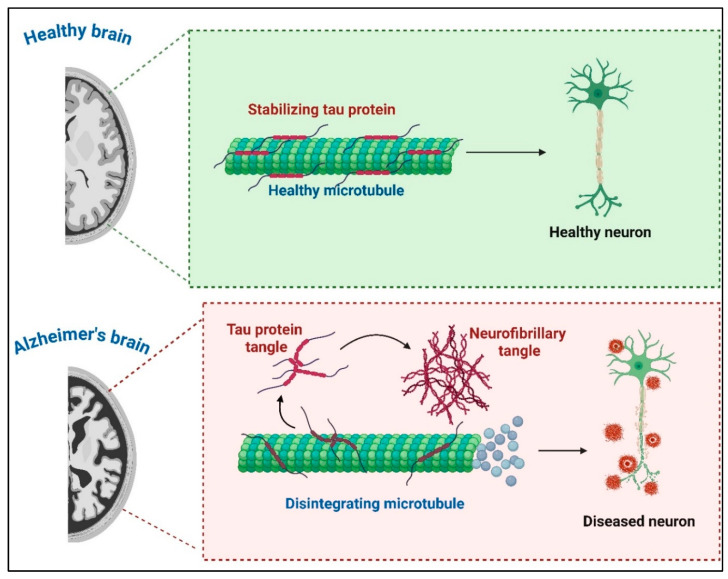
Comparison of pathological hallmarks in a healthy brain versus an Alzheimer’s brain. The healthy brain shows normal structure without deposits, while the Alzheimer’s brain exhibits amyloid-beta (Aβ) plaques, tau tangles (NFTs), brain atrophy, and chronic inflammation (Created using Biorender, https://biorender.com/kazopjs, accessed on 23 September 2025).

Alzheimer’s disease (AD) is a progressive neurodegenerative disorder characterized by cognitive decline, memory loss, and impaired daily functioning. With an aging global population, AD prevalence is increasing, affecting approximately 33–39 million people worldwide in 2025 and projected to reach 152 million by 2050, highlighting the need for innovative therapeutic strategies. The hallmarks of AD pathology include amyloid-beta (Aβ) plaques, neurofibrillary tangles (NFTs) formed by hyperphosphorylated tau, and chronic neuroinflammation (Figure 1) [1,2,3].

Traditional therapeutic approaches, such as cholinesterase inhibitors, provide symptomatic relief but fail to address the underlying pathological mechanisms of the disease [4]. Immunotherapy, particularly antibody-based therapies, has gained attention for targeting Aβ and tau. Monoclonal antibodies (mAbs) have recently achieved FDA approval (aducanumab, lecanemab, donanemab), their limited brain penetration and risks such as ARIA emphasize the need for improved modalities [5,6].

Nanobodies (Nbs) or single-domain antibodies derived from camelid heavy-chain antibodies offer engineerable features such as high solubility, modularity for multivalent constructs, and microbial producibility, making them attractive candidates for preclinical AD studies [7,8]. This review examines the role of Nbs in AD immunotherapy, focusing on their mechanisms of action and translational potential.

## 2. Nanobodies: Structure and Advantages

**Figure 2:**
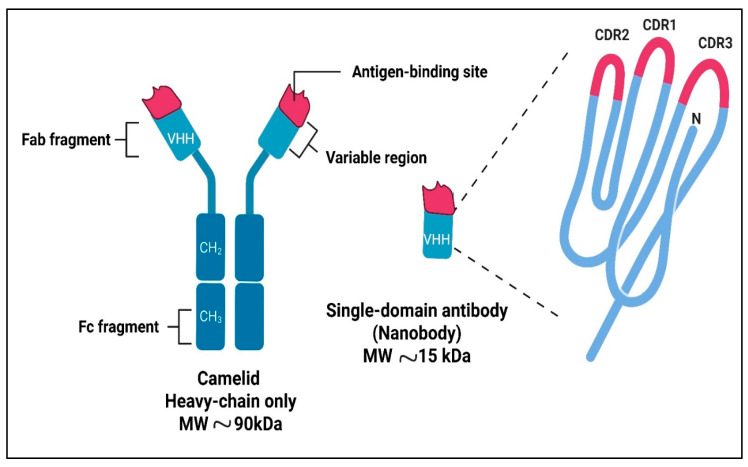
Schematic representation of single domain antibody (VHH), showing the three complementarity-determining regions (CDR1-CDR3) highlighted as protruding loops (Created using Biorender, https://biorender.com/7l8d0ji, accessed on 23 September 2025).

Nbs are small antigen-binding fragments that typically range from approximately 12 to 15 kDa [9] (Figure 2). They are derived from the variable domain of heavy-chain-only antibodies found in camelids, with similar single-domain antibodies present in cartilaginous fish [10]. Key characteristics of Nbs include high solubility, the ability to access recessed epitopes, and suitability for engineering into bispecific or multivalent constructs [11].

Compared with full-length immunoglobulin G (IgG), Nbs offer advantages such as excellent tissue penetration, reduced immunogenicity due to diminished Fc-mediated effector functions, and greater flexibility for multivalent or bispecific constructs [12]. These attributes make Nbs especially appealing for targeting the central nervous system (CNS), where large antibodies struggle due to steric access and limited brain penetration [12,13].

The compact size of Nbs enables enhanced tissue penetration within many tissues; however, they do not passively cross the intact blood–brain barrier (BBB) at therapeutically relevant levels and instead require additional engineering, such as receptor-mediated transport modules, similarly to other biologic formats [14]. Nbs exhibit high solubility, thermal stability, and resistance to aggregation, making them suitable for long-term storage and administration [15]. Additionally, they can be engineered for enhanced specificity, conjugated to imaging agents, or fused with other proteins to improve functionality [16]. These properties make nanobodies ideal candidates for targeting AD-related pathologies.

## 3. Mechanisms of Nanobody-Based Immunotherapy in Alzheimer’s Disease

Nb-based immunotherapy in AD focuses on three primary pathological targets: Aβ aggregates, tau pathology, and neuroinflammation [16].

### 3.1. Amyloid-Beta Aggregates

Nbs targeting Aβ species have demonstrated efficacy by binding to toxic oligomers, blocking oligomerization and fibril formation, and neutralizing neurotoxicity [17]. Nbs targeting Aβ species have demonstrated efficacy by binding to toxic oligomers, blocking oligomerization and fibril formation, and neutralizing neurotoxicity in preclinical models. Neurotoxic soluble Aβ oligomers are increasingly recognized as a key driver of AD pathogenesis and a clinically validated target for conventional antibody therapy [18]. Recent advances in AD immunotherapy have led to the regulatory approval of several amyloid-beta–targeting mAbs, such as aducanumab, lecanemab, and donanemab. These agents are administered intravenously, demonstrate modest brain penetration and clinically meaningful amyloid reduction on PET imaging, but are associated with amyloid-related imaging abnormalities and require intensive safety monitoring [5]. In contrast, nanobody candidates targeting amyloid-beta remain at the preclinical stage, where their small size and engineering flexibility allow diverse delivery strategies (intranasal, intrathecal, and receptor-mediated BBB shuttles) and multivalent formats that have shown robust plaque and oligomer reduction in animal models [6]. Together, these approved mAbsprovide a clinical benchmark against which the future efficacy and safety of amyloid-beta-directed nanobody therapeutics can be evaluated.

Specific Nbs (e.g., A4, E1, V31-1) inhibit the aggregation of Aβ oligomers, thereby preventing progression to mature fibrils implicated in plaque formation. Some Nbs can enzymatically degrade Aβ at secretase cleavage sites, thereby offering a direct reduction in amyloid burden [18]. Delivery of Nbs constructs fused with BBB shuttle domains, such as FC5, which targets a sialo glycoprotein receptor on brain endothelium to induce receptor-mediated transcytosis, enables efficient brain uptake [19]. This modality has demonstrated reductions in brain Aβ levels, improvements in hippocampal volume, and normalization of cerebrospinal fluid (CSF) Aβ ratios in AD model mice. Multifunctional Nb conjugates with imaging agents (e.g., gadolinium) provide diagnostic capabilities for noninvasive tracking of amyloid pathology [20].

As summarized in Table 1, FDA-approved amyloid-beta antibodies define current clinical standards in terms of efficacy and safety, whereas nanobodies are at an earlier, preclinical stage but may offer next-generation engineering possibilities.

To make this contrast explicit, a comparative Table 2 that summarizes the main properties of FDA-approved anti-amyloid antibodies and representative nanobody-based candidates. It includes (i) molecular format and target epitope; (ii) route and regimen of administration (for antibodies) or delivery strategy (for nanobodies, such as AAV-mediated expression, receptor-mediated transcytosis, or direct CNS delivery); (iii) main efficacy readouts (clinical endpoints for approved antibodies, or behavioral, pathological, and biomarker outcomes in animal models for nanobodies); and (iv) key safety considerations, including rates and management of ARIA for approved antibodies and any reported toxicity signals in nanobody studies.

Framing nanobody work within this clinical context highlights both the opportunities to build on established amyloid-lowering strategies and the substantial translational gap that remains before nanobody constructs can enter human trials.

### 3.2. Tau Pathology

Tau proteins aggregate into paired helical filaments, which form neurofibrillary tangles, thereby driving neurodegeneration. Nbs that recognize critical amyloidogenic sequences of tau (e.g., PHF6 core) inhibit tau aggregation and propagation, as demonstrated by work using VHH Z70, which reduces tau spreading in mouse models [24,25]. These Nbs can be delivered via viral vectors such as lentivirus or adeno-associated virus (AAV) for sustained intracerebral expression, offering a gene therapy approach to reduce tau pathology. Imaging probes targeting phosphorylated tau using nanobodies facilitate early detection of tauopathy [26].

### 3.3. Neuroinflammation

Nanobodies targeting inflammatory mediators (e.g., TNFR1, CXCL10, TREM2-related pathways) have shown preclinical promise, but no clinical testing has been performed in AD [22]. Crossing the BBB remains a significant hurdle for brain therapeutics. Nbs exploit receptor-mediated transcytosis (e.g., via the transferrin receptor and insulin-like growth factor receptor) and adsorptive-mediated transcytosis to enter the brain [14]. FC5 is a pioneering Nb that binds the α (2,3)-sialo-glycoprotein receptor on brain endothelial cells, triggering clathrin-mediated endocytosis and enhancing BBB crossing [27]. These receptor-mediated transcytosis and physical opening approaches are not unique to Nbs but are particularly compatible with Nb scaffolds because of their small size and modularity, which facilitates incorporation into multivalent or multi-specific constructs [28]. Physical methods, such as ultrasound combined with microbubbles, transiently open the BBB to enhance nanobody delivery [29]. Alternative administration routes, such as intranasal, intrathecal, and intracerebroventricular delivery, directly target the CNS, resulting in higher local nanobody concentrations [30]. Gene therapy via AAV vectors encoding nanobodies provides a strategy for sustained brain exposure [31].

### 3.4. Translational Approaches and Challenges

Nb-based imaging agents have been developed to detect pathological Aβ and tau species with improved target specificity and rapid clearance, offering higher contrast and reduced toxicity compared to conventional antibodies (Table 3) [32]. For example, the Nb pa2H labeled with technetium-99m shows brain uptake that correlates with amyloid burden in AD models [33]. Nbs detecting retinal Aβ oligomers have been used to develop minimally invasive retinal assays that may support early AD diagnosis in preclinical and exploratory clinical settings. Therapeutically, Nanobodies inhibit toxic aggregation, promote clearance, and can be coupled to effector molecules or enzymes to degrade pathological proteins [34,35]. Preclinical studies in mouse models report reversal of hippocampal atrophy and normalization of several AD-relevant biomarkers following experimental Nb-based interventions, highlighting therapeutic potential yet to be confirmed in human trials [36,37]. Clinical translation will depend on optimizing delivery, half-life, and multifunctionality while minimizing immunogenicity [38].

The following table structure summarizes nanobody constructs relevant to AD, organized by target, model system.

## 4. Nanobody-Based Drug Delivery Systems

**Figure 3:**
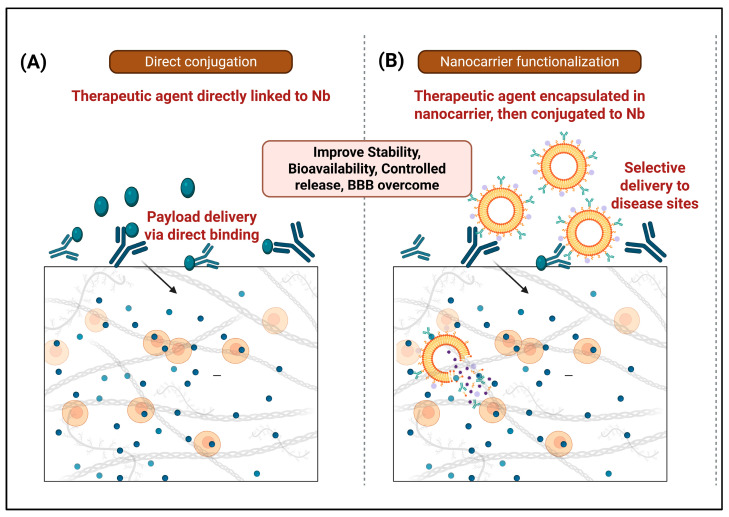
Nb-based Drug Delivery Systems. This figure illustrates two primary approaches for Nb-based drug delivery. (**A**) Direct Conjugation: Therapeutic agents are directly linked to Nbs, enabling specific payload delivery via direct binding. (**B**) Nanocarrier Functionalization: Therapeutic agents are encapsulated within nanocarriers, which are then conjugated to Nbs for selective delivery to disease sites. Both strategies contribute to improved drug stability, bioavailability, controlled release, and the ability to overcome biological barriers, such as the blood–brain barrier, in CNS disorders (Created using Biorender, https://biorender.com/3gxby84, accessed on 23 September 2025).

In this section, nanobody-based delivery strategies developed predominantly in oncology and other non-AD indications are briefly summarized as platforms that could, in principle, be adapted for AD, while acknowledging that most examples described have not yet been tested in AD models. Nb-based drug delivery systems can be broadly categorized into two principal approaches (Figure 3) [41]. The first approach involves the direct conjugation of therapeutic agents or toxic molecules to Nbs, exploiting their high specificity and affinity for targeted delivery. This strategy enables precise neutralization or payload delivery via direct binding interactions with Nb. The second approach centers on the functionalization of nanocarriers, such as liposomes, polymeric nanoparticles (NPs), and other nanostructures, with Nbs to enhance their targeting capabilities [42]. In this method, therapeutic agents are first encapsulated or incorporated into nanocarriers, which are subsequently conjugated to Nbs to facilitate selective delivery to disease-relevant sites [43]. This approach improves drug stability, bioavailability, and controlled release kinetics. Together, these strategies highlight the versatility of Nb-based platforms in optimizing therapeutic delivery, particularly in overcoming biological barriers such as the BBB in CNS disorders [44].

### 4.1. Direct Injection of Nanobodies

Direct injections may optimize therapeutic outcomes and efficacy while leveraging the beneficial small size of Nbs [45,46]. A common matrix protein in malignancies, collagen is a suitable target for immunotherapies applied locally. By using the collagen-binding ectodomain of murine leukocyte-associated immunoglobulin-like receptor-1 (LAIR) to generate specific Nb variants, Nbs are tailored for treatments targeting collagen within cells [45]. To reduce systemic toxicity and localize the therapeutic effect, these modified Nbs are subsequently fused with Interleukin-2 (IL-2), a cytokine known to activate immune cells [45,46]. Similarly, a delay in tumor growth may result from the combination of small-format IL-2 immunocytokines with high-affinity Nbs that target the tumor-specific Extra domain-B of fibronectin (EIIIB), which is present in the tumor extracellular matrix. These immunocytokines have effects on tumor growth that are comparable to those of untargeted IL-2 when administered intravenously. On the other hand, better survival outcomes are achieved with intratumoral administration, which relies on the affinity for EIIIB [47]. Additionally, Escherichia coli bacteria with a type III protein secretion system may be able to engineer Single-domain antibodies (sdAbs) for injection into human cells, thereby bypassing the requirement that the Abs pass through the plasma membrane, a significant obstacle when targeting intracellular proteins for therapeutic purposes [46]. Two particular sdAbs: Variable single-domain antibody targeting amylase (Vamy) and Variable single-domain antibody targeting green fluorescent protein (GFP), used for intracellular tracking or delivery (Vgfp), which target amylase and GFP, respectively, are used in the engineering of these sdAbs [47]. These intratumoral and intracellular delivery examples illustrate the versatility of Nb formats but do not directly address AD; they are therefore presented as conceptual precedents rather than established CNS therapies.

### 4.2. Nanobodies Conjugated to the Drug and Toxin

The majority of Nbs must be coupled with a toxic load or another effector function since they lack innate therapeutic activity. Whether the conjugation is a single effector domain or a drug-containing nanocargo, the conjugated Nbs are used for drug delivery in various applications [48,49]. Conjugation of Nbs to enzymes or toxin molecules can modestly increase hydrodynamic radius and circulation time, but substantial and sustained half-life extension typically requires dedicated strategies such as Fc-fusion, albumin binding, or PEGylation [38]. Accordingly, pharmacokinetic evaluation and immunogenicity profiling are essential components of Nb–effector fusion development before any consideration of clinical translation [50]. As a result, the construct’s ability to transport its load to tumors or sick tissues improves. There are two methods for conjugation: chemical conjugation and cloning in an expression vector by fusing the Nb gene with a hazardous protein [51,52]. Because the Nbs contain multiple lysines, the effector moiety’s conjugation to the Nb, mostly to lysine residues, may be heterogeneous in chemical conjugation. If a Lys in the CDR reacted, the reaction could block the CDR’s access to the antigen, reducing or eliminating the Nbs’ ability to recognize it. In a different tactic, the addition of a single cysteine to the Nb’s C-terminal end enables site-directed conjugation of a hazardous load far from the paratope, thereby minimizing disruption of antigen binding [53]. To connect chelators and nuclides, the C-terminal end of Nbs has recently been modified by Sortase A [54]. In any case, following conjugation, the antigen-binding qualities need to be verified. Accordingly, pharmacokinetic evaluation and immunogenicity profiling are essential components of Nb–effector fusion development prior to any consideration of clinical translation [55]. One of the most effective non-viral gene transfer methods, polyethyleneimine (PEI), is limited in its in vivo use due to several issues, including non-specific cell binding, interaction with blood components, and relatively high cytotoxicity [56]. In a work by Saqafi et al., a branched polyethyleneimine polymer (25 kDa) was attached to MAL-PEG3500-NHS (bi-functional polyethylene glycol molecules) to create a nano-carrier system based on PEI derivatives. Three distinct molar ratios of 10, 20, and 30 (polyethylene glycol: polyethylene imine) were used to create this chemical. The copolymer composed of PEI and polyethylene glycol was attached to the anti-HER2 nanobody as a targeting agent. The impact of these modifications on the cytotoxicity, physicochemical characteristics, cell absorption, and gene transfer efficiency of PEI polymers was evaluated as follows. Compared to unconjugated PEI, PEGylated PEI copolymers were less cytotoxic in vitro [44,57].

### 4.3. Nanobodies Attached to Nanocarriers

Nano-sized drug carriers, with diameters less than 200 nm, are also used in drug delivery systems. One of the most innovative approaches in pharmaceutical technology and medicine has been the development of nanoscale drug-delivery vehicles. Inorganic, magnetic, and polymeric NPs are among the various drug delivery strategies developed using NPs [58]. By lowering the effective dose, these systems can boost a drug’s bioavailability, shield it from oxido-reduction and enzymatic processes, and lessen its potential immunogenicity [59]. Toxic substances that are packaged and delivered can prevent harm and adverse effects on healthy tissues. They can also dissolve hydrophobic medications in lipidic bilayers, such as liposomes, or hydrophobic cores, such as micelles. Because of the NPs’ delayed but sustained drug release, more drug can be administered in a single dose, and the frequency of administration will decrease [60]. When such nanocarriers are functionalized with Aβ- or tau-specific Nbs and combined with BBB-crossing strategies, they may provide a modular route to targeted nanobody delivery in AD [61].

### 4.4. Nanobody-Based Immunotoxin

Nb-based immunotoxins combine molecular targeting precision with cytotoxic function, enabling selective intervention against key pathological proteins in AD. These engineered chimeric proteins link single-domain antibody fragments, which penetrate the BBB more efficiently, to toxin moieties designed to disrupt pathogenic protein aggregation or aberrant neuronal signaling [35]. Recent work has shown that Nb targeting tau and amyloid precursor protein (APP) domains fused with Pseudomonas exotoxin fragments can effectively reduce toxic aggregates in cellular and animal AD models. This targeted cytotoxicity addresses challenges inherent in conventional therapies, such as off-target effects and limited brain penetration, providing a strategic advantage for precise neurotherapeutics [17]. EGFR dysregulation is increasingly recognized as a key feature of AD pathophysiology, and EGFR-specific Nb immunotoxins offer a novel approach to modulating this pathway and mitigating neuroinflammation. Biparatopic humanized Nb constructs improve binding avidity and therapeutic potential, demonstrating robust efficacy in experimental systems [62]. Advances in recombinant protein engineering, including α-sarcin ribotoxin-linked Nb platforms and trimeric/bispecific designs, enhance delivery efficiency and therapeutic potency. These developments position Nb-based immunotoxins as promising candidates for future theranostic applications that integrate disease-specific targeting with diagnostic imaging in AD [63].

### 4.5. Nanoparticles or Liposomes-Conjugated Nanobodies

**Figure 4:**
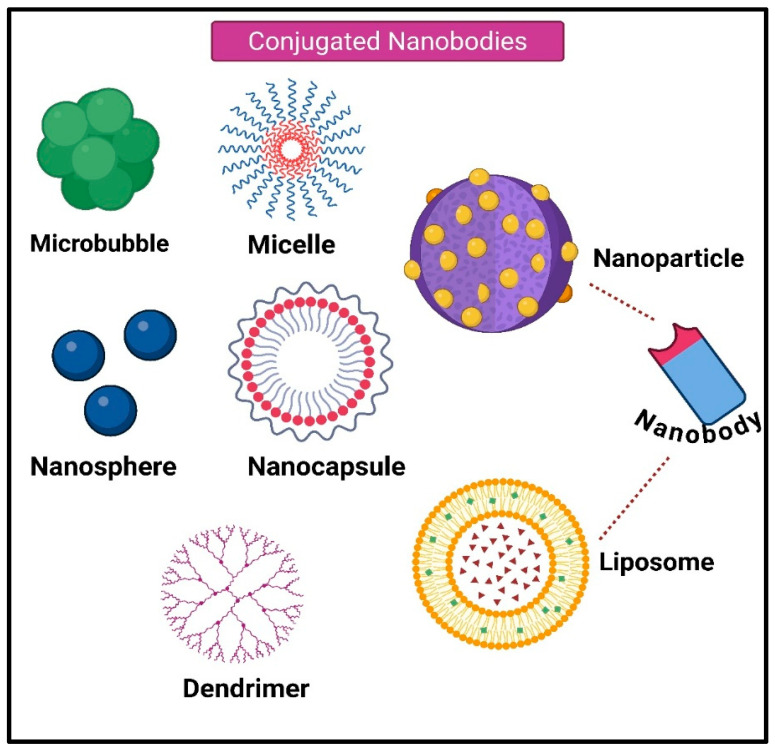
Nanocarriers Functionalized with Nanobodies (Nbs) The figure displays various types of nanocarriers including Microbubbles, Nanospheres, Micelles, Nanocapsules, Dendrimers, Nanoparticles, and Liposomes that can be conjugated with Nanobodies. This conjugation strategy leverages the high specificity of Nbs to target these drug-loaded nanocarriers to specific disease sites, enhancing drug stability and selective delivery. (Created using Biorender, https://biorender.com/aceszcl, accessed on 23 September 2025).

Liposomes have been regarded as a functional drug-carrier system since Bangham discovered them in the 1960s. Their appearance and properties are strikingly comparable to those of cellular membranes [63,64]. Given the significance of size in tumor targeting, liposomes can be made in a wide variety of sizes, from 100 to 400 nm. Numerous candidates are currently undergoing preclinical examination or clinical application, marking significant advancements over the years [65]. Liposomes’ exterior chemical variations can help Nbs, or any other protein form, targeted systems, which will eventually cause encapsulated liposomes to accumulate in tumor tissues [66,67]. In summary, Nb-liposome systems have the potential to enhance therapeutic efficacy and are suitable for combination therapy (Figure 4). Conjugating Nbs that target the hepatocyte growth factor receptor (MET-Nbs) to PEGylated liposomes at different densities between 20 and 800 Nbs per liposome is one example. Adjusting the MET-Nb density increases the specificity of the NPs toward their intended cellular target and decreases interactions with phagocytic cells [68]. In ex vivo human blood, a MET-Nb density above 300 Nbs per liposome results in a two-fold increase in interactions with phagocytic cells compared to nontargeted liposomes. Tumor-associated macrophages (TAMs), as brain-resident macrophages (microglia and border-associated macrophages), are crucial regulators of the neuroinflammatory environment in AD. These cells represent intriguing targets for halting neurodegeneration and modulating disease progression. Liposomal drug delivery systems have been developed to overcome the BBB, enabling the effective transport of therapeutic agents into the AD brain. For example, multifunctional liposomes can be engineered with brain-penetrating peptides and ligands targeting amyloid-beta (Aβ) or tau pathology, facilitating enhanced BBB crossing and targeted delivery to affected regions. These liposomes thus enable suppression of pathological signaling pathways and reduction in oxidative stress involved in AD pathogenesis [69]. Similarly, natural extracellular vesicles (EVs) hold promise as versatile, biocompatible drug carriers for AD therapy, owing to their ability to deliver therapeutic cargo across neural cell populations. Protein ligase-mediated covalent attachment of targeting moieties, such as Aβ- or tau-binding peptides and antibodies, allows EVs to accumulate selectively within AD-affected cells. Experimental models have shown that EVs can improve the therapeutic delivery of agents such as anti-inflammatory drugs and enzyme inhibitors at low doses, enhancing cognitive function and mitigating amyloid and tau aggregation [70]. Single-walled carbon nanotubes (SWNTs) also offer a novel neuroprotective approach in AD by restoring impaired autophagy pathways. Functionalized SWNTs can reverse defective autophagy flux by modulating mTOR signaling and lysosomal proteolysis, thereby promoting the clearance of protein aggregates characteristic of AD pathology. At low concentrations, SWNTs exhibit cytoprotective effects with minimal toxicity, opening avenues for their use as therapeutic nanocarriers for the management of neurodegenerative diseases [71]. Compared with conventional therapeutics, nanotechnology-based nanocarriers have improved pharmacokinetics and pharmacodynamics, reduced cytotoxicity, increased stability, and higher drug entrapment efficiency [72,73].

#### 4.5.1. Microbubbles

It has been reported that Nb-microbubble (μB) conjugates have been developed as a novel molecular tracer [69]. The lipid μBs containing streptavidin were site-specifically linked to the biotinylated anti-eGFP (cAbGFP4) and anti-VCAM-1 (cAbVCAM1-5) Nbs. Fluorescence microscopy was used to establish the selective binding of eGFP to μB-cAbGFP4 and the fast flow binding of VCAM-1 by μB-cAbVCAM1-5. Both in vitro and in vivo demonstrations of the use of VCAM-1-conjugated μBs as a novel molecular ultrasonic contrast agent have been reported [74]. It was also suggested that certain drugs may be encapsulated in μBs to achieve gradual release at the tumor site.

#### 4.5.2. Micelles

With the hydrophilic head regions in contact with the surrounding solvent and the hydrophobic single-tail regions sequestered in the micelle center, which can range from 10 to 100 nm depending on composition and concentration, a micelle is an aggregate of amphiphilic block molecules dispersed in aqueous solution [75]. Micelles are frequently used as delivery systems for hydrophobic medications that are challenging to transport in the bloodstream. Micelles help make these hydrophobic medications soluble and stable when diluted. Through the EPR effect, their nanoscale size enables an effective accumulation in malignant tissues [76]. A long circulation time for drugs or particles is required for optimal EPR effect. This can be achieved by attaching the pharmaceuticals to carrier surfaces or by coating the tiny drugs with PEG. The last tactic, therefore, suggests that attaching a targeting moiety (such as an antibody, scFv, or Nb) to the micelle surface will enhance the accumulation of carriers in the target region and promote the absorption of specific medications. When certain Nbs are coupled to the micelle surface, a tailored drug delivery mechanism is created, which promotes the internalization of pharmaceuticals that are transported [48].

#### 4.5.3. Dendrimers

Dendrimers are branching, monodisperse structures that range in size from 3 to 20 nm [77]. Targeting moieties can be coupled with dendrimers to functionalize their surface. To facilitate drug distribution, functional molecules can be encapsulated within the dendrimer’s multifunctional core. For specific uses, drug molecules, such as paclitaxel, can also be attached to the dendrimer’s exterior. To optimize pharmacokinetics and biodistribution, DOX was recently conjugated to carboxyl-terminated poly(amidoamine) (PAMAM) dendrimers and evaluated in a lung metastasis model [78].

#### 4.5.4. Nanospheres

The drug can be encapsulated within an aqueous or oily core of a nanosphere, a delivery vehicle composed of a spherical polymeric matrix ranging in size from 1 to 100 nm. The drug is then released gradually as it circulates through the bloodstream. To extend the half-life and facilitate Nbs binding for targeted therapy, the nanosphere’s surface can also be PEGylated [79,80].

#### 4.5.5. Nanocapsules

Nanoparticle medications are contained within the cores of nanocapsules, which are nanoscale shells ranging in size from 10 to 1000 nm. A polymeric barrier keeps the medications from escaping the environment [81,82]. Numerous industries, including medicine, food enhancement, nutraceuticals, and self-healing materials, use nanocapsules for drug delivery. The targeted distribution of chemicals to specific tissues is now the most appealing application. Precise targeting can be achieved by attaching monovalent, bivalent, or even trivalent Nbs to the surface of this delivery system [83,84]. Such multimeric Nbs coupled to nanocapsules have not yet been published, but they are nevertheless a very appealing material with considerable promise for further study.

### 4.6. Albumin Nanoparticles-Conjugated Nanobodies

Albumin NPs are another highly effective delivery system. The most prevalent plasma protein, albumin, plays a crucial role in several vital processes. Additionally, albumin is safe and biocompatible, and Muller et al. suggested using albumin NPs as a drug delivery method [78]. Several researchers were motivated to develop a safe medication delivery system using such albumin NPs [85,86].

### 4.7. Gene Therapy-Associated Nanobody

Viral vector-assisted gene therapy has emerged as a key tool in both practical medicine and the fundamental biological sciences. Currently, viruses including herpes simplex virus, adenovirus, and adeno-associated virus (AAV) are preferred for this purpose [87,88]. The well-known vectors LV and AAV [89,90] can be used to introduce genes, including those encoding Nbs, into host cells to create intracellular Nbs (also known as intrabodies). The most researched model for immunotherapy and gene delivery is the LV. Unfortunately, it remains challenging to transfer the desired genes from lentiviral particles to the target cells, such as tumor cells or antigen-presenting cells (APCs), following in vivo delivery [91]. Virion buildup in the liver and spleen is typically the outcome of administering wild-type AAV and LV vectors. An intriguing method was devised by Dropulic [91], who constructed a modified LV vector using an envelope glycoprotein derived from VSV-G, adorned with Nb DC2.1, which was binding-defective but fusion-competent. This Nb targets dendritic cells (DCs), which are essential for the activation of antigen-specific T lymphocytes along with macrophages. Through genetic engineering, *Lactobacillus*, a naturally occurring component of the gut microbiota, can be engineered to express VHH against norovirus. When given orally to germ-free mice, the Nb VHH 1E4 efficiently neutralizes the GII.17 norovirus. Their potential as oral Nb delivery vectors for passive immunization against norovirus infections is highlighted by the fact that these modified *lactobacilli* maintain their neutralizing activity in the intestines for at least 10 days [92]. Nbs can be administered using a similar method to prevent necrotic enteritis in chickens brought on by *Clostridium perfringens*. Nbs that neutralize *C. perfringens* NetB toxins can be effectively produced and secreted by recombinant *Limosilactobacillus reuteri* [93]. A promising method for managing and preventing PRRSV infections is the development of Nb6-pFc, a Nb fused with porcine IgG Fc (Fcγ), which blocks the highly contagious porcine reproductive and respiratory syndrome virus (PRRSV) from replicating in susceptible cells via Fcγ-receptor-mediated endocytosis [94]. AAVs have been developed into vectors for gene therapy and are generally considered safe for humans. The targeting of human CD4+ cells, such as primary human peripheral blood mononuclear cells and purified human CD4+ T lymphocytes, is enhanced by genetically engineered AAV2 capsid proteins, VP1 and VP2, which harbor Nbs with high affinity for the human CD4 receptor [95]. Additionally, combining adenoviral-mediated gene transfer with antibody-based targeting offers a powerful approach for precise in vivo gene delivery in AD. Engineering sdAbs into the chimeric fiber of adenoviral vectors composed of fibritin, adenovirus serotype 5 fiber, and an AD-relevant sdAb enables brain cell-specific tropism. This strategy enhances targeted delivery of therapeutic genes to neurons and glial cells affected by AD pathology, improving gene transfer efficiency and therapeutic outcomes. Such capsid modifications reduce off-target effects and hold promise for advancing gene therapy in AD [96].

## 5. Nanobody Delivery in AD

Amyloid burden, neuroinflammation, synaptic function, and cognition are among the pathologies of AD that can be positively impacted by the long-lasting, brain-wide synthesis of VHH-B9 that AAV vectors can deliver into the CNS of AD model mice [97]. Multivalent Nb PNBILs are a new and innovative approach to treating AD by reducing oxidative stress and amyloid-β (Aβ) aggregation. The PNBIL reduces AD symptoms in mouse models by recognizing and inhibiting Aβ aggregation, as well as facilitating microglia-mediated clearance, after being modified with Aβ and interleukin-1β (IL-1β) fragments [98]. Aβ aggregates are also a target for treatment in AD. In an in vivo study, the brain-penetrating anti-amyloid Nb fusion protein FC5-mFc2a-ABP decreased brain Aβ levels, increased the Aβ42/40 ratio in cerebrospinal fluid, and improved neurological tests [99]. Similarly, this disease is characterized by the aggregation of tau protein [100]. Pathological Tau buildup is reduced by VHH Z70, which is expressed in the brains of tauopathy mouse models [35]. A different strategy uses inhibitory Nbs that target tau aggregation by grafting sequences (VDW, W3, and WIW) onto a Nb scaffold. These Nbs bind tau, VQIINK, and VQIVYK amyloid-driving sequences [100]. Moreover, Nbs from an alpaca vaccinated with T-cell receptor’s extracellular domain have strong affinity and epitope mapping potential for disease research and diagnostics; decreased sortilin-related receptor with A-type repeats (SORLA) levels in AD require accurate detection [24].

Even though Nbs have been used in many novel ways to treat neurodegenerative illnesses, most of these molecules are still in preclinical testing. Through mutagenesis, one preclinical model discovered an Nb that targets the mouse transferrin receptor (mTfR). Nb62 was one of the variations that bonded to the chimeric receptor. Nb62 and neurotensin (NT), which cause hypothermia in the brain, were united by researchers. External measurements of animals given Nb62-NT intravenously showed dose-dependent hypothermia, a sign of brain penetration (Figure 4) [101]. By directly affecting body temperature, this model provides a precise evaluation of brain target engagement, offering a non-invasive preclinical investigation that may lead to therapeutic applications. AAV vectors, for instance, carry antibody-coding sequences into CNS cells for in situ Nb synthesis, improving therapeutic efficacy and facilitating Nb delivery across the BBB [102].

## 6. Emerging Nanobody-Based Therapeutic Strategies

Many of the bispecific, trispecific, CRISPR-based, and AI-assisted approaches described in this section have so far been developed primarily with conventional antibody scaffolds; analogous design principles can be applied to nanobody formats in AD, but this remains largely at the conceptual and preclinical stage [22,23]. All things considered, Nbs are more than just monoclonal Ab replacements; their special qualities expand the range of biotherapeutic uses, such as molecular imaging [97], microscopy targeting and visualization [103], pharmaceutical diagnostic testing, such as recombinant human interferon α2b [104], and adjustable drug targeting systems [105,106]. Nbs have spurred neuromodulation and regenerative medicine studies. Inflammation is a factor in many nervous system disorders, including multiple sclerosis (MS), which can be treated by reducing inflammation and promoting myelin regeneration. Nbs that target CXCL10 and TNFR1 have been tested for the treatment of MS. In MS models, TROS, an anti-TNFR1 Nb, prevents inflammation and protects neurons and myelin. By blocking CXCL10-CXCR3 binding, Nb 3Nb12’s action against CXCL10 inhibits cell chemotaxis and may have applications in regenerative medicine [107]. Neuromodulation modifies synaptic strength to influence clusters of neurons. Inhibitory neurotransmitters activate G-Protein-Coupled receptors (GPCRs) and anion channels, whereas excitatory neurotransmitters activate cation channels and the appropriate GPCRs. Researchers employed a conformation-specific Nb in this neuromodulator method to study β2-adrenoceptor activation. The active version of β2AR is the particular target of this Nb80. Consequently, Nb80-EGFP (Enhanced Green Fluorescent Protein) migrates from the cytoplasm to the plasma membrane upon β2AR activation. This is another new use, still theoretical for research on neuromodulating medicine, although it is challenging to achieve in vivo [17,107].

Due to the intricate pathophysiology of AD, which involves tau, Aβ, neuroinflammation, and vascular dysfunction, single-target mAbs are limited, necessitating bispecific antibodies that target multiple pathways simultaneously [108]. Aβ/Triggering Receptor Expressed on Myeloid Cells 2 (TREM2) and Aβ/tau are two examples of bispecific antibodies that target two antigens together to enhance delivery and efficacy [108]. To increase brain delivery by 8–10 times, the designs incorporate IgG-like formats or nanobodies that leverage knobs-into-holes engineering in conjunction with BBB transporters (e.g., Transferrin Receptor 1 [TfR1]) [109]. Despite these encouraging preclinical findings across multiple AD models, nanobody-based interventions for AD have not yet entered human efficacy trials, and their status should therefore be regarded as experimental.

Aβ, tau, and neuroinflammation are among the multifactorial pathologies of AD that are targeted by bispecific antibodies, which are designed to bind two antigens [82]. Although none are currently on the market for AD as of May 2025, preclinical and early clinical evidence indicate great potential [110]. Their design, mechanics, preclinical developments, clinical trials, BBB penetration tactics, and future directions are all thoroughly examined in the sections that follow.

### 6.1. Bispecific Antibody Platform

The design and use of platforms such as CrossMab, Knobs-into-Holes, Genmab DuoBody, BiTE, WuXiBody, SMABody, YBODY, and FIT-Ig differ. Knobs-into-Holes is scalable but only mildly immunogenic, whereas CrossMab guarantees high purity but necessitates sophisticated manufacture. BiTEs have a brief half-life, which restricts their application in the CNS. Due to their stability and long half-life, IgG-like platforms (CrossMab, Knobs-into-Holes) are recommended for developments enable scalable, stable bispecific antibodies for AD therapy. These Bispecific antibodies for AD are being revolutionized by emerging methods that enhance delivery, efficacy, and specificity. Although these developments tackle the complex pathophysiology of AD, they encounter technological and legal obstacles [23].

### 6.2. Trispecific Antibodies

Trispecific antibodies have synergistic effects because they target three different antigens. In P301S/APP mice, a trispecific antibody binding Aβ, tau, and TREM2 decreased pathology by 40%, reducing AT8-positive neurofibrillary tangles and thioflavin-S-stained plaques, while also increasing Morris water maze performance by 30% [111]. Nevertheless, the complexity of manufacturing results in low yields (less than 50% purity) and high costs (approximately 1.5–2 times those of bispecific antibodies), and immunogenicity risks necessitate Fc modification [111]. However, delivery problems are exacerbated by the larger size of trispecific and bispecific antibodies.

### 6.3. CRISPR-Based Gene Editing

By correcting APP mutations in APP/PS1 mice and lowering Aβ production by 35% and CSF Aβ42 levels by 6 months by AAV delivery, CRISPR/Cas9 in conjunction with anti-Aβ/anti-tau bispecific antibodies [111]. Plaque formation was reduced to 5xFAD mice when PSEN1 was silenced [112]. Translation is limited by regulatory barriers, high vector costs ($500,000–$1 Million per Patient), and off-target editing (5–10% in neural cells) [113]. Standardized administration and increased Cas9 specificity are essential. AI-Driven Antibody Design: AI maximizes binding affinity and antigen selection. AI-optimized anti-Aβ/anti-TfR1 antibodies produced a 12-fold increase in brain absorption in Tg2576 mice, removing 20% more plaques than controls. At the same time, machine learning algorithms predicted Aβ/TREM2 and Aβ/tau couples with a Kd of 1–3 nM [114]. However, AI necessitates validation across a variety of populations (e.g., APOE4 variations) and large datasets (>10,000 structures), which are frequently lacking for AD antigens [115]. Incorporating single-cell RNA sequencing may improve accuracy.

### 6.4. Advanced Systems of Delivery

In APP/PS1 mice, anti-Aβ/anti-CD98hc antibodies reduced hippocampus Aβ with fewer off-target effects than TfR1 by utilizing the large neutral amino acid transporter to produce an 8-fold increase in brain uptake [116,117]. In *Tg2576* animals, lipid NPs containing anti-Aβ/anti-TREM2 antibodies enhanced delivery by a factor of 12, resulting in a 25% decrease in CSF p-tau181 [118]. Scalability is hindered by stability issues and high production costs ($100,000/kg) [118]. Although it requires specialized equipment, the combination of focused ultrasound with anti-Aβ/anti-tau antibodies decreased plaque formation by 30% in 5xFAD mice [113].

### 6.5. Combination Treatments

Bispecific antibodies will be used in conjunction with gene therapies, small-molecule inhibitors, and neurodegenerative medicines in future trials (e.g., NCT05744401). Precision medicine is made possible by plasma p-tau217 tests, which stratify patients [93]. But there are still issues with scalability, regulatory barriers to gene therapy, and safety (including ARIA and cytokine release) [109]. To enable individualized, successful AD therapeutics, future directions include scalable nanoparticle platforms, accurate CRISPR base editing, cost-effective trispecific designs, and the integration of multi-omics AI.

## 7. Preclinical and Clinical Progress of Nanobody-Based Therapeutics

The approval of caplacizumab has increased interest in domain Abs, indicating that regulators may approve camelid-based VHH domains [119,120]. For patients with advanced multiple myeloma who are refractory or have relapsed after at least four lines of therapy, the FDA also authorized Ciltacabtagene autoleucel (Carvykti), a second-line medication, as a CAR-T-cell therapy [121]. A trivalent anti-TNFα called ozonoralizumab was approved in Japan for the treatment of poorly controlled RA [122]. Nevertheless, despite the quick expansion of Ab research, only 11 of the 675 current Ab initiatives in clinical development are domain Abs [119]. For a growing number of clinical studies, it is crucial to identify niche areas where Nbs can outperform other biologics [119]. A future where Nb-based therapy overcomes current obstacles is also suggested by ongoing clinical trials and research that emphasize Nbs as promising biological agents, with the possibility of patient-targeted therapeutic stratification through non-invasive imaging [123]. For instance, creative methods can overcome delivery obstacles. Therapeutic drugs may be transported across the BBB by Nb188, an anti-human TfR VHH. Nb188 may cross the CNS when united with neurotensin, something that the neuropeptide cannot do on its own. Moreover, it has been demonstrated that the production of heterodimeric Abs by combining the anti-β-secretase 1 (BACE1) 1A11 Fab with Nb62 or Nb188 effectively lowers Aβ1–40 levels in the brain, underscoring its potential as a therapeutic drug delivery system to the CNS [124].

### 7.1. Bispecific Antibodies

Bispecific antibodies are categorized into two groups: (a) pathology-targeting bispecific antibodies, which engage AD-related targets (like Aβ/tau, Aβ/TREM2) for synergistic effects; and (b) BBB-shuttling bispecific antibodies, which combine CNS-active antibodies with brain-penetrating modules (like TfR1, CD98hc) to improve delivery [125]. Bispecific antibodies outperform mAbs in AD mouse models.

#### 7.1.1. Pathology Targeting Bispecific Antibodies

In 5xFAD animals, anti-Aβ/anti-TREM2 antibodies enhanced microglial plaque uptake, decreased amyloid PET signals, and enhanced Morris water maze performance [114]. Levels of p-tau181 and CSF neurogranin were decreased [126]. In 3xTg-AD mice, anti-Aβ/anti-tau antibodies improved CA1 neuron long-term potentiation by reducing plaques and NFTs (immunohistochemistry) [127]. Anti-Aβ/anti-APOE4 antibodies decreased plasma neurofilament light (NfL) in APP/PS1 mice, thereby reducing synaptic damage [128]. Anti-Aβ/anti-CD33 and anti-Aβ/anti-NLRP3 antibodies boosted Aβ absorption and decreased inflammation (TNF-α, IL-1β) in Tg2576 and 3xTg-AD mice [129,130].

#### 7.1.2. Bispecific BBB-Shuttling Antibodies

In Tg2576 mice, anti-Aβ/anti-TfR1 antibodies reduced CSF Aβ42 by 30% and produced a 10-fold increase in brain absorption [114]. Using the large neutral amino acid transporter, anti-Aβ/anti-CD98hc antibodies enhanced brain delivery eight times in APP/PS1 mice while having fewer off-target effects than TfR1 [131]. In APP/PS1 mice, anti-Aβ/anti-BACE1 antibodies decreased soluble Amyloid Precursor Protein β (sAPPβ), which in turn decreased Aβ formation [132]. Bispecific antibodies targeting pathology, such as anti-Aβ/anti-tau, reduced pathology by 30% in 3xTg-AD mice; however, glycoengineering is necessary to mitigate immunogenicity risks [129]. These results demonstrate the potential of bispecific antibodies to treat various aspects of AD; however, issues such as immunogenicity and scalability necessitate further optimization.

Preclinical research has demonstrated that bispecific antibodies are more effective than mAbsin several AD mouse models [22]. An anti-Aβ/anti-TREM2 antibody was administered to 5xFAD mice, which improved their performance in the Morris water maze, reduced amyloid PET signals, and increased microglial plaque uptake [133]. There was a decrease in CSF neurogranin, a marker of synaptic loss, and in tau pathology (p-tau181) [127]. The combination of Aβ-APOE4-neutralizing anti-Aβ/anti-APOE4 antibodies decreased synaptic damage in APP/PS1 mice while lowering neurofilament light (NfL) in plasma [128]. The anti-Aβ/anti-tau antibody administered to 3xTg-AD mice improved CA1 neuron long-term potentiation and decreased plaque counts and NFTs (immunohistochemistry) [134]. Anti-Aβ/anti-CD33 antibody-treated Tg2576 mice exhibited increased Aβ uptake and decreased pro-inflammatory TNF-α levels [135]. In 3xTg-AD mice, the combination of anti-Aβ/anti-NLRP3 antibodies inhibited inflammasome activation, resulting in lowered tau phosphorylation and IL-1β [136]. Anti-Aβ/anti-BACE1 antibodies decreased soluble Amyloid Precursor Protein β (sAPPβ) and Aβ production in APP/PS1 mice [130]. Anti-tau/anti-GSK3β antibodies decreased AT8-phosphorylated tau in P301S mice, and their motor performance on the rotarod test improved [120]. Pathology decreased in P301S/APP animals treated with anti-Aβ/anti-TREM2 and anti-tau/anti-GSK3β, suggesting possible synergistic effects [137]. These studies demonstrate that bispecific antibodies can address multiple aspects of Alzheimer’s disease [133].

Early-stage clinical trials (Phase I/II) test bispecific antibodies for the treatment of AD. These trials assess medication safety in conjunction with pharmacokinetics and biomarkers, including glial fibrillary acidic protein (GFAP), amyloid PET, CSF Aβ42, and plasma p-tau217 [133]. As of May 2025, no bispecific antibodies are available on the market for the treatment of AD, despite several candidates showing promise [138]. AL002 (Anti-TREM2/Anti-Aβ): Alector’s product AL002 works in two ways: it utilizes Aβ to remove plaques and TREM2 to activate microglia [133]. With different doses (15, 40, or 60 mg/kg biweekly) over 96 weeks, the Phase II trial (NCT04592874, INVOKE-2, intended n = 265, MMSE 22–30, 50–85 years) did not demonstrate significant effects on amyloid PET or other CSF biomarkers (e.g., p-tau, neurogranin). There have been reports of ARIA-E and ARIA-H, with APOE4 homozygotes having a greater incidence. There were no discernible effects on clinical outcomes similar to those observed with CDR-SB. It was decided to end the extension trial (NCT05744401) {Phase II Extension Study of AL002. 2025. Available online: http://clinicaltrials.gov (accessed on 20 May 2025)}. Anti-Aβ/Anti-TfR1: Trontinemab (RO7126209), a bispecific antibody targeting both Aβ and TfR1, is currently in Phase II development (NCT04639050, Brainshuttle AD trial) [139]. Compared with traditional antibodies, preclinical research showed markedly improved brain uptake (e.g., a 4–18-fold increase in brain exposure). According to the interim data from the Phase Ib/IIa study (NCT04639050), there has been a notable decrease in amyloid plaque, as well as early and notable decreases in CSF and plasma biomarkers, including total tau, phosphorylated tau 181 (p-tau181), p-tau217, and neurogranin. For trontinemab, a Phase III clinical trial is planned. A significant difficulty is the complexity of manufacture; other challenges include the need for reliable biomarkers and the potential release of cytokines from immune-targeting antibodies. mAbs or small compounds utilized in combination therapy (e.g., NCT04241068) are the subject of studies [140]. When paired with plasma p-tau217 and GFAP stratification, APOE4 status serves as the primary moderating factor that improves trial outcomes [141,142].

#### 7.1.3. BBB Bypass

Only 0.1% to 0.3% of the given dosage reaches the brain due to the BBB’s restriction of antibody transport [143]. Bispecific antibodies, which attach to BBB transporters, enhance the entry of antibodies into the CNS [22]. However, due to their size, trispecific and bispecific antibodies encounter comparable or even greater difficulties [22]. BBB-shuttling bispecific antibodies and other methods increase CNS delivery. TfR1 and CD98hc: Through transcytosis (Kd = 2 nM), anti-Aβ/anti-TfR1 antibodies enhanced brain absorption tenfold in Tg2576 mice, resulting in a 30% decrease in CSF Aβ42 [116,144]. By leveraging strong BBB expression and minimizing off-target effects, anti-Aβ/anti-CD98hc antibodies increased uptake 8-fold in APP/PS1 mice [117,145]. In preclinical studies, trontinemab (anti-Aβ/anti-TfR1) demonstrated a 12-fold increase in uptake [117].

#### 7.1.4. Alternative Strategies

In 5xFAD mice, anti-Aβ antibody uptake was boosted threefold by RVG peptides, which target nicotinic acetylcholine receptors [146,147]. In APP/PS1 mice, insulin receptor-mediated transport resulted in a 5-fold increase in uptake [118]. In Tg2576 animals, lipid NPs containing anti-Aβ/anti-TREM2 antibodies enhanced delivery by a factor of 12, lowering CSF p-tau181 [23]. Materials based on silicon are being investigated [113]. In 5xFAD mice, focused ultrasonography using anti-Aβ/anti-tau antibodies temporarily opened the BBB, reducing plaque formation and restoring spatial memory [113]. In APP/PS1 mice, adeno-associated virus (AAV) vectors reduce plaques by maintaining antibody production for six months [125]. Clinically feasible, these tactics, particularly TfR1, CD98hc, and NPs, need to be optimized to strike a balance between safety and effectiveness. For AD and related dementias, such niches are likely to include brain imaging, targeted delivery modules within larger biologics, and highly modular multispecific constructs, with clinical validation still pending.

## 8. Challenges and Future Directions

Despite the strong preclinical evidence, the adoption of nanobody-based therapeutics still faces significant challenges. Addressing these challenges could transform clinical translation and become an effective intervention for AD treatment. A key obstacle restricting the application of nanobodies in AD is the BBB, which severely limits delivery of systemically administered biologics; Nb constructs therefore require active delivery strategies (e.g., receptor-mediated transport, focused ultrasound, or intrathecal/intranasal routes) rather than relying on passive diffusion [148]. The use and application in humans remain unresolved, as long-term safety concerns, as well as scalability and regulatory acceptance limitations, remain unresolved [14,25,27]. More recently, other transport mechanisms, such as nanoparticle and lipid-based strategies and insulin receptor-mediated methods, have significantly improved brain delivery, with further optimization and validation still to be completed [114,116,117,145].

One major constraint with nanobodies is the rapid systemic clearance. Studies are currently ongoing to overcome this pharmacokinetic barrier and extend the half-life of nanobodies via PEGylation, Fc fusion, or the use of albumin-binding domains [36,38]. Although nanobodies lack Fc-mediated effector functions and can be humanized, robust head-to-head clinical data comparing their immunogenicity to full-length mAbs are limited, and repeated dosing in a chronic indication such as AD could still elicit anti-drug responses that must be carefully monitored [149]. This highlights the importance of careful engineering and humanization. In addition, current animal models do not fully reflect the complex nature of human AD pathology, which limits their ability to predict clinical outcomes [1,2].

Alongside these existing challenges, new approaches are emerging that could change the field. Bispecific and trispecific nanobody constructs targeting combinations of Aβ, tau, and neuroinflammatory pathways may help overcome the limitations of single-target treatments [108,127,132]. Gene-editing methods, such as CRISPR/Cas9, enable earlier intervention in disease processes. Artificial intelligence is now being used to enhance antibody design, including the optimization of binding affinity, antigen selection, and interactions with BBB transporters [112,114]. Although these technologies show great potential, they also bring new regulatory, ethical, and manufacturing challenges that must be carefully considered before they can be used in clinical settings.

Manufacturing and regulatory hurdles remain significant concerns. Creating multifunctional antibodies, nanoparticle conjugates, and viral vectors at a clinical scale is a complex and costly process. Gene therapies also entails high production costs and strict regulatory oversight. Using standardized biomarkers, such as plasma p-tau217 and GFAP, helps select patients and measure clinical outcomes [128,141,147]. New mAbs, including lecanemab and donanemab, have set new standards; however, their limited effectiveness and safety indicate that better treatments are still needed, and nanobody platforms should be viewed as promising but as yet unproven alternatives that require rigorous comparative evaluation [22,23]. Nanobody platforms appear promising due to their modularity and ability to reach the brain; however, more direct comparison studies are needed.

Continued advancement in the field requires the systematic integration of multifunctional nanobody platforms with advanced delivery systems and emerging genetic and computational technologies. This progress must be supported by comprehensive regulatory frameworks and sustained interdisciplinary collaboration among molecular engineers, neuroscientists, clinicians, and policymakers. These coordinated efforts are critical for translating nanobody-based strategies from laboratory research to clinically practical therapeutic and diagnostic applications for Alzheimer’s disease.

## 9. Conclusions

Nanobody drugs represent a promising therapeutic potential for AD rather than an established therapy. They provide distinct modular advantages over conventional antibodies that can be exploited to improve CNS targeting and brain exposure when combined with appropriate delivery strategies. The preclinical evidence is robust, demonstrating the capacity to target amyloid-β, tau, and neuroinflammation, in addition to serving as imaging tools and making them promising theranostics. Several challenges still need to be addressed, including finding ways for nanobodies to cross the BBB, enhancing their stability in the body, mitigating immune reactions, and overcoming manufacturing and regulatory hurdles. Moving forward, combining nanobody technology with new delivery methods, multi-specific engineering, gene editing, and AI-based antibody design could help target multiple disease pathways and support precision medicine. Close teamwork across different fields and thorough clinical testing will be crucial to turn nanobodies from a promising idea into effective treatments and diagnostic tools for Alzheimer’s disease. Overall, current evidence supports nanobodies as versatile experimental tools and emerging therapeutic candidates whose ultimate clinical value in AD will depend on overcoming BBB, pharmacokinetic, immunogenicity, and manufacturing challenges demonstrated in human studies.

## Figures and Tables

**Table 1:** Key differences between FDA-approved amyloid-beta antibodies and Alzheimer’s disease nanobody candidates.

Feature	FDA-Approved Antibodies	Nanobodies
Size (kDa)	~150	~15
BBB penetration	Low	Enhanced via engineering/receptors
Delivery	Intravenous, Intrathecal	Intravenous, intrathecal, intranasal, receptor-mediated (preclinical)
Efficacy evidence	Human trials (PET, cognition)	Robust in rodents, not yet in humans
Clinical limitations	ARIA, infusion reactions	Limited clinical data; lower immunogenicity in preclinical work
Modifiability	Moderate	High (multivalent, conjugatable)

**Table 2:** Nanobody Therapeutics Relative to FDA-Approved Anti-Amyloid Antibodies.

Therapeutic/Modality	Target/Epitope and Forma	Development Stage/Trial or Model	Administration/Delivery Strategy	Key Efficacy Readouts	Key Safety/ Limitations	Reference
Aducanumab (IgG1 mAb)	Aggregated Aβ (plaques and some oligomers); full-length IgG	Phase 3 EMERGE/ENGAGE; approved for early AD in US (subsequently discontinued commercially in some regions)	IV infusion every 4 weeks	Amyloid PET reduction; small effect on cognitive decline in one phase 3 trial	High ARIA-E/H incidence; intensive MRI monitoring; controversies over benefit–risk balance	[5]
Lecanemab (IgG1 mAb)	Aβ protofibrils; full-length IgG	Phase 3 Clarity AD; approved for early AD	IV infusion every 2 weeks (with subcutaneous formulation in development)	Amyloid PET reduction; statistically significant but modest slowing of cognitive and functional decline	ARIA-E/H risk requiring MRI monitoring; infusion reactions; access and cost considerations	[5]
Donanemab (IgG1 mAb)	Pyroglutamate Aβ (*N*-terminally modified plaque epitope); full-length IgG	Phase 3 TRAILBLAZER-ALZ 2; approved for early AD	IV infusion every 4 weeks, with treatment discontinuation once sufficient amyloid clearance is achieved	Robust amyloid PET reduction; ~25–30% slowing of clinical decline on composite scales	ARIA-E/H risk with boxed warning; MRI monitoring; treatment limited to early stage and amyloid-positive patients	[21]
Anti-Aβ nanobodies (general class)	Soluble Aβ oligomers, protofibrils, or fibrillar epitopes; single-domain VHH formats ± multivalent or Fc-fused constructs	Preclinical in vitro and in vivo studies (AD mouse models, ex vivo human tissue)	Intravenous delivery with BBB shuttle, AAV-mediated CNS expression, direct CNS delivery, or other experimental routes	Reduction in soluble oligomer load, plaque burden, or tau pathology; rescue of synaptic and behavioral deficits in models	No human safety data in AD; potential immunogenicity and off-target effects require formal toxicology and PK/PD studies	[22]
Other AD-relevant nanobody constructs	Targets include tau oligomers, BACE1, microglial receptors, and neuroinflammatory mediators	Preclinical proof-of-concept studies in cell culture and animal models	AAV-based intrabodies, intraparenchymal or intrathecal delivery, systemic delivery with BBB engineering	Modulation of tau aggregation and spreading, reduced amyloid production, or dampened neuroinflammation in models	Translation to humans will require optimization of delivery, specificity, and immunogenicity	[6,23]

**Table 3:** Overview of Alzheimer’s related Nanobodies by Target and Model system.

Nanobody Target	Nanobody/ Construct	Primary Target/Epitope	Model System(s)	Outcomes	References
Amyloid-beta	Nanobody specific for soluble Aβ oligomers	Conformational epitopes on soluble Aβ oligomers	In vitro oligomer assays; Aβ-overexpressing mouse model	Reduced synaptotoxicity; preservation of LTP; binding to oligomeric and plaque Aβ in brain	[20]
Tau	Conformational nanobodies against tau oligomers	Pathological tau oligomer conformers	Human AD and primary age-related tauopathy brain samples; cell-based aggregation assays	Specific labeling of pathological tau; interference with aggregation/seeding in vitro	[24]
BACE1	Anti-BACE1 VHH delivered by AAV	BACE1 catalytic domain	Transgenic AD mouse models with AAV-mediated	Reduced BACE1 activity; lowered Aβ production; improvements in synaptic and cognitive endpoints	[39]
Neuroinflammation-modulating nanobodies	Plaque-targeted nanobodies fused to immunomodulatory effectors	Aβ fibrils or microglial receptors	Transgenic AD mouse models; neuroinflammation assays	Enhanced plaque clearance; modulation of microglial activation; impact on cognitive outcomes	[40]
BBB	Transferrin receptor-binding nanobody shuttles	Endothelial receptors mediating transcytosis (e.g., TfR1)	Rodent models; in vivo brain exposure and imaging studies	Enhanced brain uptake of fused cargo; defined relationship between dose, exposure, and CNS distribution	[14]
Diagnostic	Retinal Aβ oligomer-binding nanobodies	Aβ1-40 and Aβ1-42 oligomeric forms	APP/PS1 mice; blood and retinal tissue	Detection of Aβ oligomers in retina and blood prior to overt brain pathology; support for minimally invasive ocular diagnostics	[34]

## Data Availability

No new data were created or analyzed in this study. Data sharing is not applicable to this article.
